# Diabetes-associated differentially expressed genes as prognostic biomarkers and therapeutic targets in endometrial cancer: a comprehensive molecular analysis

**DOI:** 10.3389/fonc.2025.1591040

**Published:** 2025-10-30

**Authors:** Ting Zhang, Ruiqing Sun, Xuejun Lian, Changyu Wang, Yuping Li, Kang Liu

**Affiliations:** ^1^ Department of Gynecology, Affiliated Chenggong Hospital of Xiamen University, Xiamen, Fujian, China; ^2^ Department of Burn and Plastic Surgery, Affiliated Chenggong Hospital of Xiamen University, Xiamen, Fujian, China

**Keywords:** UCEC, diabetes, DM-DEGs, biomarkers, prognostic mode

## Abstract

**Background:**

Uterine corpus endometrial carcinoma (UCEC) is a prevalent malignancy increasingly observed in patients with diabetes mellitus. A comprehensive understanding of the intricate molecular interplay between diabetes and UCEC is crucial to develop effective prognostic and therapeutic strategies. This study aims to elucidate the relationship between diabetes and UCEC by identifying diabetes-related differentially expressed genes (DM-DEGs) and to establish a prognostic model to enhance clinical outcomes.

**Methods:**

Transcriptomic data sourced from The Cancer Genome Atlas (TCGA) was analyzed alongside diabetes-associated genes from GeneCards. Differential expression analysis revealed 931 differentially expressed genes (DEGs) in the training cohort and 1,206 DEGs in the validation cohort. By intersecting these DEGs with diabetes-related genes, we pinpointed 186 DM-DEGs, which were further subjected to Gene Ontology (GO) and Kyoto Encyclopedia of Genes and Genomes (KEGG) pathway enrichment analyses.

**Results:**

The univariate Cox analysis identified 17 DM-DEGs that demonstrated significant prognostic relevance. Through protein-protein interaction assessments, a LASSO regression model discerning five pivotal genes (*TRPC1*, *SELENOP*, *CDKN2A*, *GSN*, *PGR*) for prognostic modeling was constructed. This model successfully stratified patients into high- and low-risk cohorts, with Kaplan-Meier survival analysis and Receiver Operating Characteristic (ROC) curve assessment confirming notable survival differentiations. A personalized nomogram, integrating clinical parameters and risk scores, exhibited robust predictive capability, yielding a C-index of 0.781. Gene set enrichment analysis (GSEA) suggested significant involvement in pathways related to glucose and lipid metabolism.

**Conclusion:**

In conclusion, our study establishes and validates a robust prognostic signature based on diabetes-related genes (DM-DEGs) for UCEC. This signature not only effectively stratifies patient risk but also delineates specific molecular pathways, such as those involving *SELENOP*, *CDKN2A*, and *PGR*, through which the diabetic milieu may drive tumor aggressiveness. These findings provide a mechanistic rationale for the diabetes-UCEC link and pave the way for developing personalized treatment strategies. Future work should focus on translating this signature into clinical practice and elucidating the precise biological roles of these DM-DEGs.

## 1 Introduction

Endometrial cancer (EC), also known as uterine corpus endometrial carcinoma (UCEC), represents a significant health concern, being one of the most prevalent gynecological malignancies worldwide (1). The economic burden associated with EC is substantial, encompassing direct medical costs, lost productivity, and the emotional toll on patients and their families (2). Current treatments mainly consist of surgery, radiation therapy, and systemic therapies (3). Although early-stage UCEC is often curable by surgery, the overall 5-year survival rate remains below 60%, primarily due to high recurrence rates and poor response to chemotherapy in advanced or high-risk cases (4). Despite advancements in understanding the molecular basis of EC, there is still a significant gap in identifying reliable prognostic biomarkers that can categorize patients by risk profiles and inform personalized treatment approaches. This study aims to address this gap by investigating diabetes-related differentially expressed genes (DM-DEGs) in UCEC, thereby providing insights into their potential roles in disease prognosis and therapeutic response.

This study focuses on the identification and analysis of DM-DEGs in UCEC. Previous research has established a link between diabetes and several types of cancer, including endometrial cancer, suggesting that metabolic dysregulation may play a role in tumor development (5, 6). Diabetes has long been recognized as a risk factor for UCEC (7), and subsequent studies have further confirmed its significance. For instance, Wise reported that women with diabetes have a 42% higher risk of developing UCEC compared to those without diabetes (8). Moreover, Harding et al. found that mortality rates are significantly increased in UCEC patients with type 1 diabetes (9). Therefore, diabetes is not only a risk factor for UCEC development but also intricately linked to its prognosis. However, the specific molecular mechanisms underlying the association between diabetes and UCEC remain poorly understood. This research uses comprehensive transcriptomic data from The Cancer Genome Atlas (TCGA) to clarify the role of diabetes-related genes in UCEC. This will provide insights into potential biomarkers for prognosis and targets for therapy. The identification of 186 DM-DEGs and their subsequent functional enrichment analyses highlight the potential of these genes in influencing cancer biology, particularly in the context of metabolic disorders. This study not only contributes to the existing body of knowledge but also underscores the importance of integrating metabolic factors into cancer research, paving the way for personalized treatment strategies in UCEC patients.

This study used a bioinformatics method to examine the gene expression differences related to diabetes in UCEC patients. The methodology included obtaining transcriptomic data, clinical information, and somatic mutation data from TCGA. A total of 540 patients were included after excluding those with incomplete clinical or follow-up information. The analysis was refined using the Limma package in R, which led to identifying DM-DEGs. This approach enabled us to integrate large-scale genomic data with clinical outcomes, and thereby identify potential prognostic biomarkers. The main objective of this research was to create a prognostic model based on diabetes-related genes and evaluate its predictive accuracy. Additionally, the study aimed to explore the biological mechanisms through functional enrichment analyses, enhancing understanding of the relationship between diabetes and endometrial cancer prognosis.

## 2 Materials and methods

### 2.1 Data acquisition

On August 20th, we acquired transcriptomic data along with relevant clinical and somatic mutation information for patients diagnosed with UCEC from TCGA (10) (https://cancergenome.nih.gov/). The dataset comprised records from 35 healthy individuals and 554 UCEC patients (11). Following the exclusion of individuals lacking complete clinical or follow-up information, we finalized a cohort of 540 patients for subsequent analysis. Detailed Inclusion and Exclusion Criteria are presented in **Table 1**. Furthermore, utilizing the GeneCards database (https://www.genecards.org/), we employed a filtering criterion encompassing protein-coding genes and diabetes-related annotations, yielding a total of 12,374 genes. To enhance the precision of our selection, we retained only the 3,111 genes with correlation coefficients exceeding the average value (**Supplementary Table S1**), which will be subjected to subsequent differential gene expression analysis. The gene expression data were normalized using the Robust Multi-array Average (RMA) method, as implemented in the limma R package (12).

**Table 1:** Inclusion and exclusion criteria.

Inclusion criteria	Exclusion criteria
Patients were pathologically diagnosed with endometrial carcinoma (UCEC).	Patients with a history of other malignancies.
Complete clinical follow-up data, including overall survival (OS) time and status, were available.	Incomplete or missing key clinical data (e.g., survival time, pathological type).
Gene expression profiles were complete and suitable for subsequent analysis.	Poor-quality gene expression data (e.g., insufficient sample size, excessive outliers).
Data were obtained from public databases (e.g., TCGA, GEO) or ethically approved clinical cohorts.	Follow-up duration was too short (e.g., less than 30 days) for meaningful survival analysis.

### 2.2 Selection of diabetes-related genes

The training and testing cohorts were developed using the TCGA dataset, where we performed a random split of the data in a 70/30 ratio. To ensure the robustness and generalizability of our model training, we employed a standard data splitting technique with random grouping implemented in the R programming language. During the grouping process, we utilized a stratified sampling method and fixed the random number seed (seed=21) to ensure the reproducibility of the results. The dataset for this investigation was randomly partitioned into two distinct groups: a training cohort comprising 70% (377 endometrial cancer patients and 24 controls) and a testing cohort consisting of 30% (163 endometrial cancer patients and 11 controls), with the number of samples from each group delineated in **Table 2**. A preliminary differential expression analysis was conducted utilizing Limma (version 3.48.3) within the R programming environment. We applied stringent screening criteria of |log2Fold change| > 1.5 and P < 0.001 to pinpoint DEGs in both the training and testing cohorts. The identified DEGs were visualized through volcano plots and heatmaps, generated using the ggplot2 R package (https://ggplot2.tidyverse.org) and the pheatmap R package (https://CRAN.R-project.org/package=pheatmap), respectively. The intersection of DEGs from both cohorts with diabetes-associated genes led to the identification of DM-DEGs (Diabetes Mellitus), which was illustrated through a Venn diagram.

**Table 2:** Number of samples from each group.

Sample type	Train group (TCGA)	Validation group (TCGA)	Total
UCEC	377	163	540
Normal	24	11	35

### 2.3 Functional enrichment analysis of DM-DEGs

To explore the potential biological implications of diabetes-related genes in endometrial cancer compared to a normal control group, we conducted gene ontology (GO) (13) enrichment analysis and Kyoto Encyclopedia of Genes and Genomes (KEGG) (14) pathway analysis on the identified genes within the overlapping set. This analysis was performed using the R package clusterProfiler. Pathways were considered statistically significant at a false discovery rate (FDR) of <0.05 and a P value of <0.05.

### 2.4 Gene prognostic model construction

In this study, we utilized the R package ‘survival’ to integrate survival duration, survival outcome, and gene expression data, employing the Cox proportional hazards model to evaluate the prognostic significance of individual genes. Genes exhibiting statistically significant variations in univariate Cox analysis were subsequently analyzed through LASSO-Cox regression (15) using the glmnet R package, which also encompassed survival duration, survival outcome, and gene expression data. To ascertain the most effective prognostic model, we implemented a 10-fold cross-validation procedure that determined the optimal lambda value to be 0.0397805821179575. Consequently, we developed a prognostic model that incorporated five genes, represented by the following equation: RiskScore = 0.0369622344921353 * CDKN2A - 0.0152533112991654 * GSN - 0.162293585605867 * PGR + 0.190782928845233 * SELENOP + 0.0536308514116534 * TRPC1.

For risk stratification, we used the maxstat R package (version 0.7-25) to determine the optimal cut-off value for the RiskScore, ensuring that the smallest group contained no less than 25% and the largest no more than 75% of the samples. The optimal cut-off value was identified as 0.189733273285035. Based on this threshold, patients were divided into high and low risk groups. We then used the survfit function from the survival R package to analyze prognostic differences between the two groups and conducted a log-rank test to assess their significance. The analysis demonstrated a statistically significant difference in prognosis, with a p-value of 1.1e-10. To evaluate the predictive performance of the model, we performed Receiver Operating Characteristic (ROC) analysis utilizing the pROC R package (version 1.17.0.1). The roc function was implemented to conduct ROC analysis at intervals of 365, 1095, and 1825 days, while the CI function was employed to estimate the Area Under the Curve (AUC) along with its confidence intervals. This methodology was similarly applied to validate the prognostic model using an independent validation cohort.

### 2.5 Validation of key gene protein expression in endometrial tissue

To further validate the expression patterns of the five key genes (CDKN2A, GSN, PGR, SELENOP, and TRPC1) identified in our prognostic model at the protein level, we utilized immunohistochemistry (IHC) staining data from the Human Protein Atlas (HPA) (https://www.proteinatlas.org/). The HPA database provides high-resolution IHC images for a wide range of human proteins in both normal and cancerous tissues. We specifically retrieved and analyzed the IHC staining results for these five proteins in normal endometrial tissue and UCEC samples.

### 2.6 Nomogram construction

In this study, we utilized the R package rms to construct a nomogram that integrates survival time, survival status, and four key characteristics of patients through Cox regression analysis. This technique assesses the prognostic relevance of these variables within a cohort of 377 individuals. The upper section of the nomogram operates as a scoring mechanism, while the lower section serves as a predictive tool. By calculating both the total score and the individual contributions of each characteristic, we successfully predicted the survival rates and recurrence probabilities for EC patients at 1, 2, 3, 5, and 10 years. The model’s predictive capabilities were further validated through calibration curves, ROC curves, Kaplan-Meier survival curves, and the C-index, affirming its efficacy in forecasting survival outcomes.

### 2.7 GSEA enrichment analysis of hub genes

We carried out Gene Set Enrichment Analysis (GSEA) to investigate the role of hub genes in influencing UCEC. The GSEA software (version 3.0) was obtained from the GSEA website (16) (http://software.broadinstitute.org/gsea/index.jsp). Based on the expression levels of hub genes, samples were categorized into high-expression groups (≥50%) and low-expression groups (<50%). We downloaded the c2.cp.kegg.v7.4.symbols.gmt subset from the Molecular Signatures Database (17) (http://www.gsea-msigdb.org/gsea/downloads.jsp) to assess pertinent pathways and molecular mechanisms. We established the minimum gene set size at 5 and the maximum at 5000, performing 1000 permutations based on gene expression profiles and phenotypic classifications. A P value of < 0.05 and an FDR of < 0.25 were deemed statistically significant.

### 2.8 Correlation analysis of tumor microenvironment and immune infiltration

We examined alterations in the tumor microenvironment (TME) of patients diagnosed with UCEC utilizing the R package “estimate” to score the TCGA-UCEC dataset based on the Stromal Score, Immune Score, and ESTIMATE Score. Subsequently, we applied the CIBERSORT methodology (18) from the IOBR package to compute scores for 22 distinct types of immune infiltrating cells in each sample. We compared immune cell infiltration between high-risk and low-risk patient groups, with results visualized through stacked plots and box plots. Moreover, we analyzed correlations among the 22 immune cell types utilizing Pearson’s correlation test, representing these correlations with a correlation matrix.

### 2.9 Mutation correlation analysis

We conducted an exhaustive analysis of the disparities in somatic mutations between high-risk and low-risk groups. The TCGA database offers exome sequencing data for nearly 30 cancer types. We employed the R package maftools for our analysis, visualizing the outcomes with the oncoplot function.

### 2.10 Validation of the expression of biomarkers

To validate the differential expression of five diabetes-related genes (TRPC1, SELENOP, CDKN2A, GSN, and PGR) in uterine corpus endometrial carcinoma (UCEC) and to investigate the influence of diabetes comorbidity, we performed quantitative real-time polymerase chain reaction (qRT-PCR). A total of 24 endometrial tissue samples were collected from the Affiliated Chenggong Hospital of Xiamen University and divided into three groups: UCEC A (UCEC+DM, n=8), UCEC B (UCEC, n=8), and Control group (n=8). Detailed clinical characteristics of the participants are summarized in **Supplementary Table S7**. The study protocol received approval from the Medical Ethics Committee of the Affiliated Chenggong Hospital of Xiamen University (Ethics No. 73JYY2025199192).

Total RNA was extracted from each sample using TRIZol reagent (Thermo Fisher, Shanghai, China) and reverse-transcribed into cDNA using the SureScript-First-strand-cDNA-synthesis-kit (Servicebio, Wuhan, China). qRT-PCR was conducted using SYBR Green qPCR Master Mix, with each reaction performed in triplicate. The housekeeping gene GAPDH served as an internal control for normalization. The primer sequences for all target genes and GAPDH are listed in **Supplementary Table S6**.

Relative gene expression was quantified using the 2^−ΔΔCt^ method, with the Control group designated as the calibrator to express results as fold changes. First, to assess basal expression differences in UCEC, the combined UCEC A and UCEC B groups (n=16) were compared against the Control group. Second, to determine the specific impact of diabetes, a direct comparison was made between UCEC A and UCEC B. A p-value of less than 0.05 was considered statistically significant.

### 2.11 Statistical analysis

All statistical evaluations and data visualizations, including volcano plots, forest plots, box plots, violin plots, scatter plots, heatmaps, Kaplan-Meier curves, and ROC curves, were executed using R software version 4.2.1 (available at https://www.r-project.org/). To identify genes associated with prognosis, we performed univariate Cox regression analysis. The Wilcoxon rank-sum test was utilized to evaluate differences in immune infiltration scores and checkpoint-related gene expression between the two risk groups. A t-test was employed to examine differential gene expression between UCEC and normal tissue samples. Furthermore, Pearson correlation analyses were conducted to investigate the interrelationships among the 22 distinct immune cell types. For the time-dependent ROC analysis, the “timeROC” package was used to assess the predictive accuracy of the risk score at 1, 3, and 5 years, and the CI function from the same package was applied to compute the 95% confidence intervals for the AUC values via bootstrap resampling. Statistical significance of the gene expression differences was assessed using the Mann-Whitney U test. All statistical tests were two-tailed, with significance levels denoted as follows: P < 0.05 was indicated by *, P < 0.01 by **, P < 0.001 by ***, and P < 0.0001 by ****. Non-significant differences were denoted as ns.

## 3 Results

**Figure 1:**
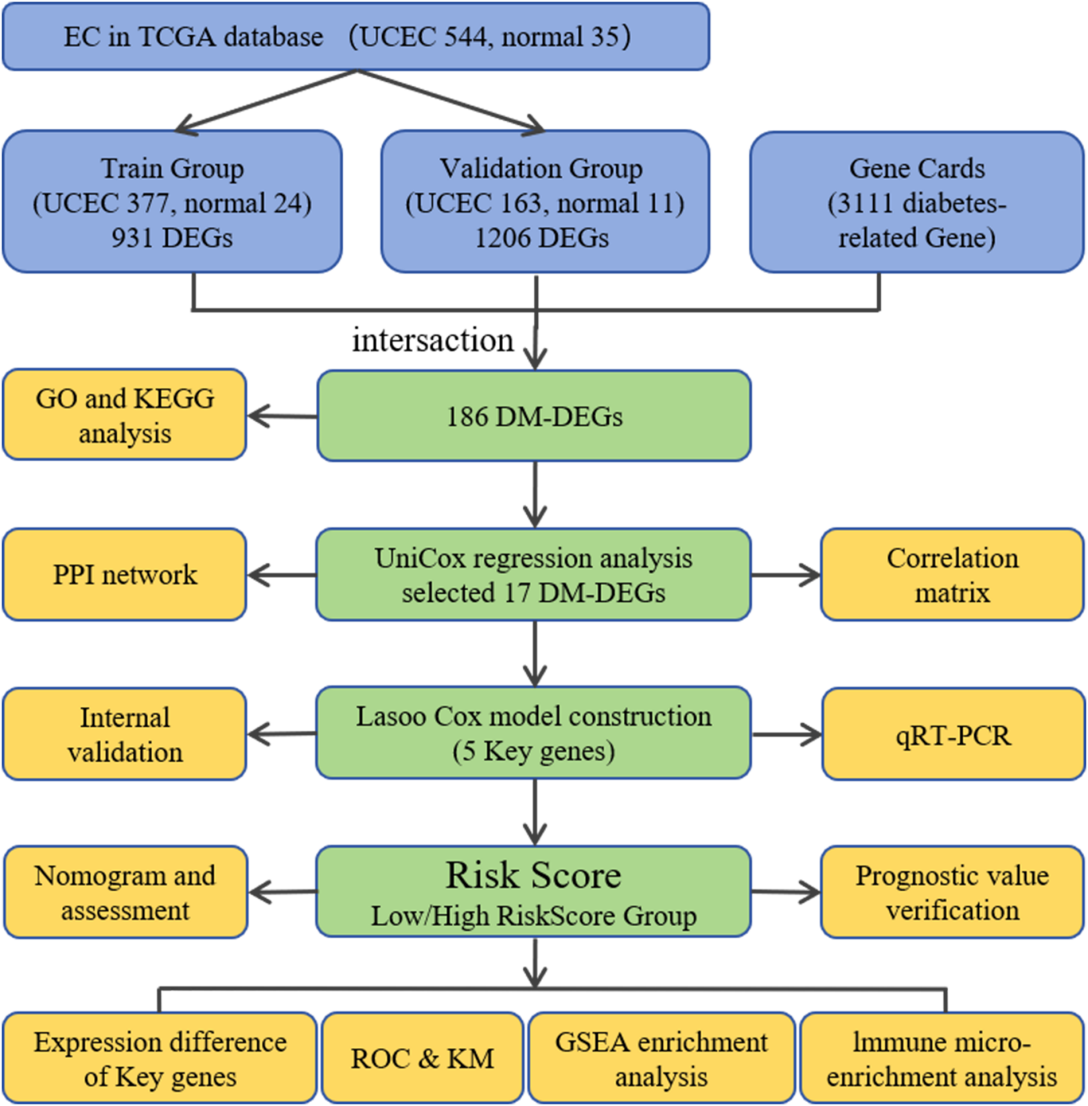
Study design flowchart.

The flow chart of this study is shown in **Figure 1**.

### 3.1 Identification of DEGs associated with diabetes mellitus in endometrial cancer

**Figure 2:**
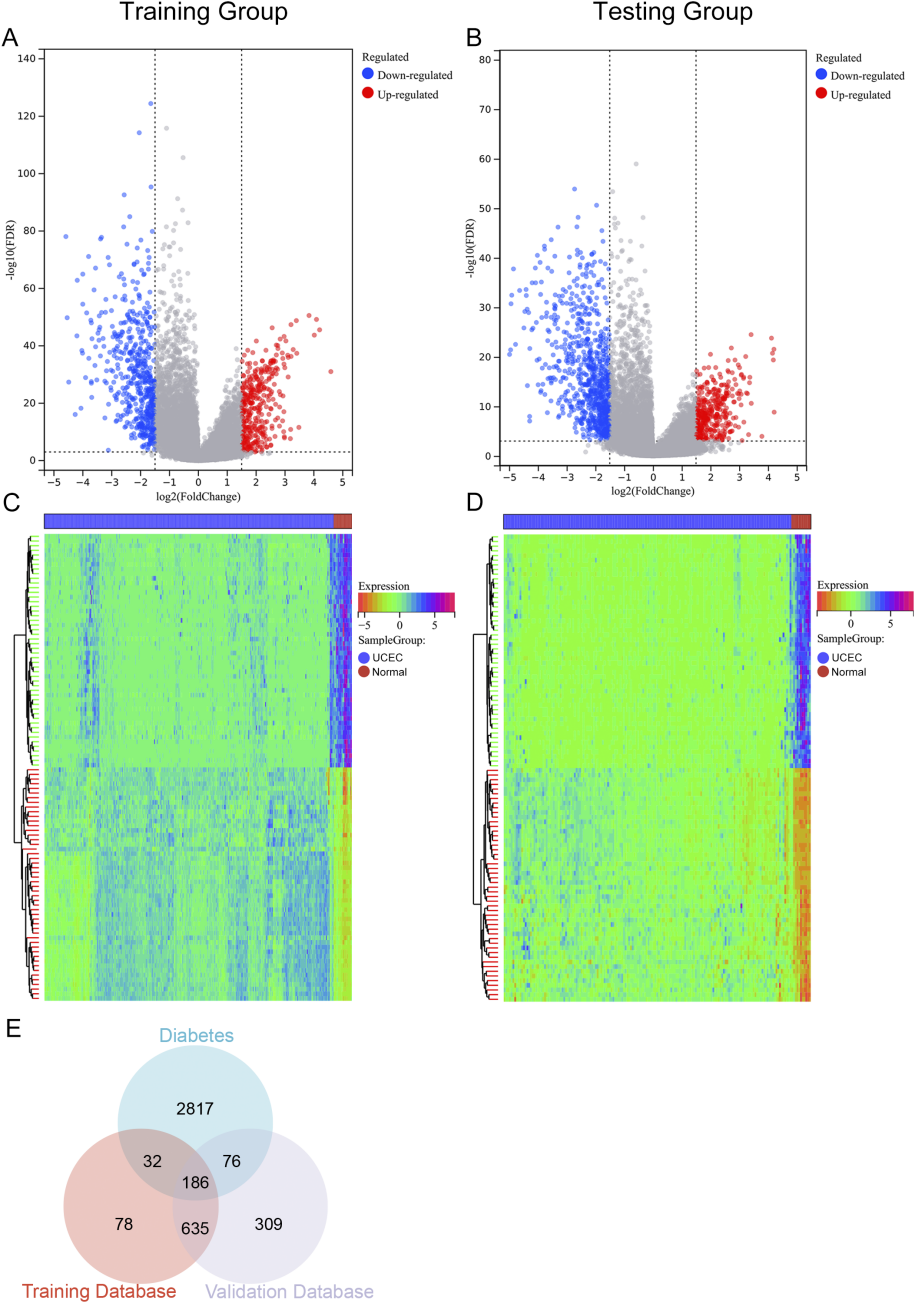
Volcano plot, heat map, and Venn diagram. **(A, B)** The volcano plots for the Training group and Testing group, with upregulated genes represented in red and downregulated genes in blue. **(C, D)** The heat maps for the Training group and Testing group. **(E)** Venn diagram illustrating overlapping genes from both groups and the diabetes database.

In the training cohort, a total of 931 DEGs were detected when comparing 377 UCEC samples to 24 control samples, with 374 genes exhibiting upregulation and 557 genes showing downregulation (**Figures 2A, C**). In the validation cohort, 1206 DEGs were identified between 164 UCEC samples and 11 control samples, comprising 397 upregulated genes and 809 downregulated genes (**Figures 2B, D**). Subsequently, by cross-referencing with 3111 diabetes-associated genes, a total of 186 DM-DEGs (**Supplementary Table S2**) were obtained (**Figure 2E**).

### 3.2 GO and KEGG enrichment analysis of DM-DEGs

**Figure 3:**
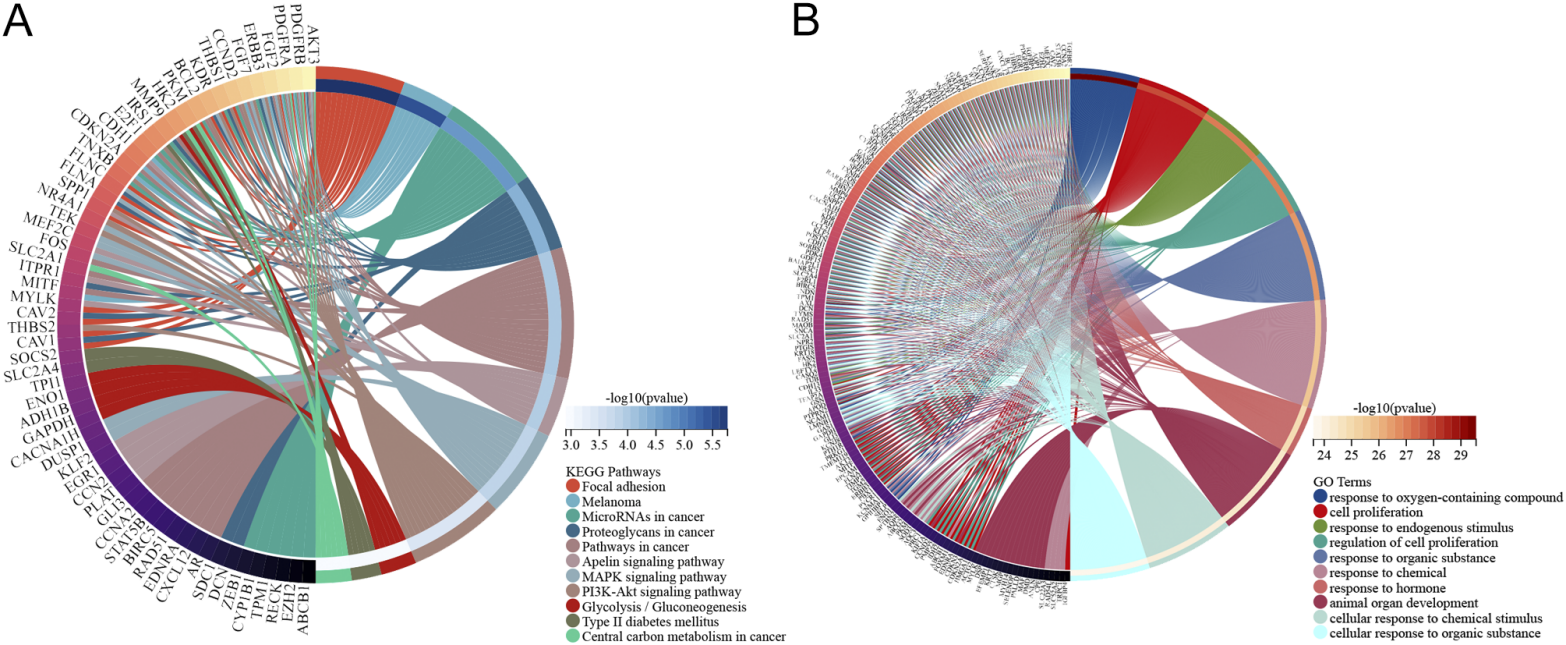
Pathway enrichment analysis of DM-DEGs. **(A)** KEGG analysis diagram of 186 DM-DEGs. **(B)** GO analysis diagram of 186 DM-DEGs.

The 186 identified DM-DEGs were enriched across 1925 GO biological functions (**Supplementary Table S3**), which included cellular responses to stress (such as responses to oxygen-containing compounds and hormonal responses), regulatory mechanisms in cellular processes (including regulation of cell proliferation and multicellular organismal process), as well as adaptation and maintenance of homeostasis in response to environmental stressors (e.g., hypoxia and low oxygen levels) (**Figure 3A**). Additionally, these genes were found to be implicated in 26 KEGG pathways (listed in **Supplementary Table S4**), encompassing Type II diabetes mellitus, central carbon metabolism in cancer, glycolysis/gluconeogenesis, cancer pathways, and the PI3K-Akt signaling pathway (**Figure 3B**).

### 3.3 Identification and correlation analysis of prognostic-related DEGs

**Figure 4:**
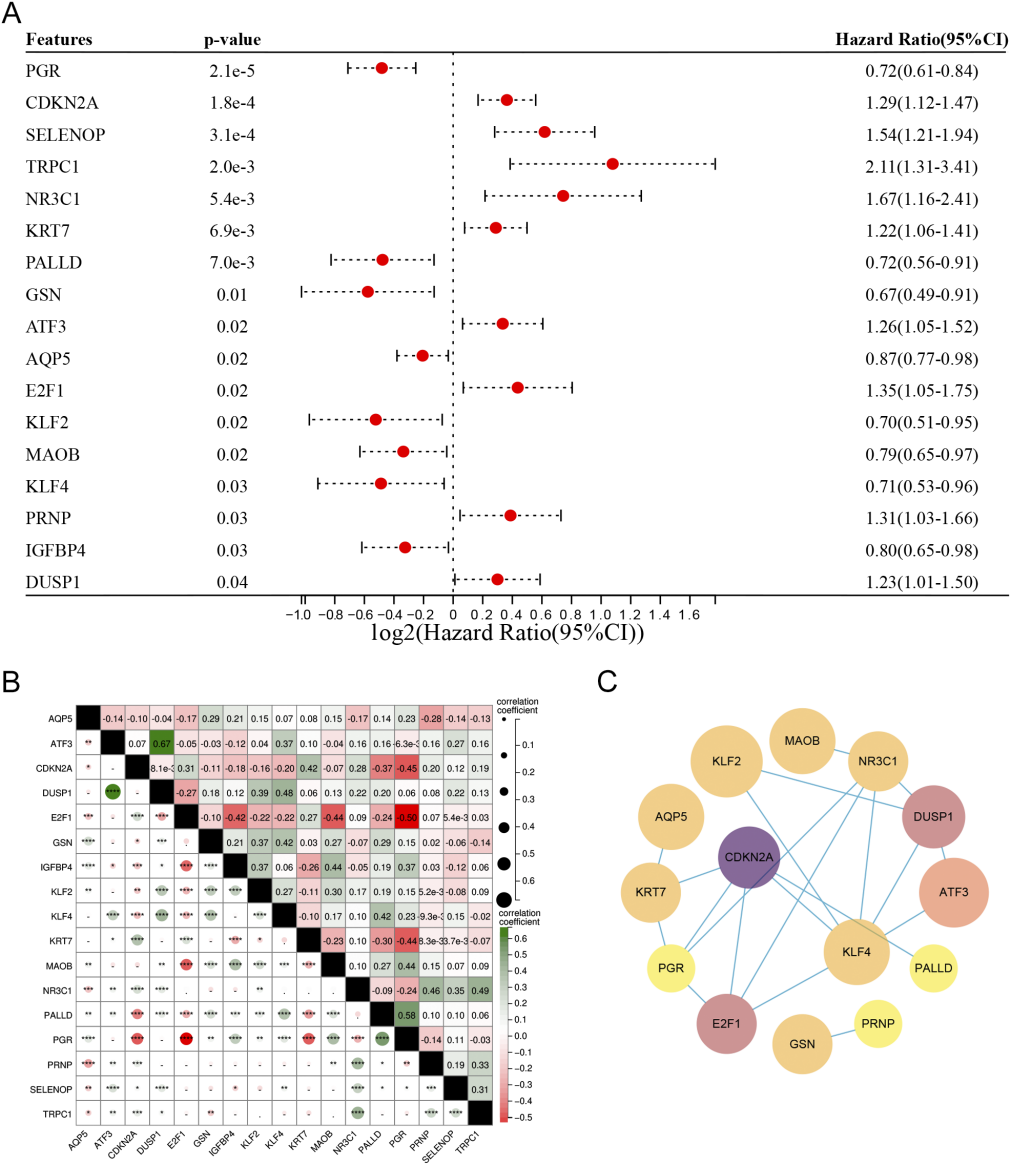
**(A)** Univariate Cox regression analysis of the 186 DM-DEGs, identifying 17 genes significantly associated with prognosis. **(B)** Correlation matrix for the 17 DM-DEGs. **(C)** PPI networks and key modules constructed via STRING and Cytoscape, featuring 20 edges and 17 nodes.

Among the 186 DM-DEGs, univariate Cox regression analysis confirmed that 17 DM-DEGs were significantly correlated with the prognosis of EC patients (P < 0.05) (**Figure 4A**). A correlation analysis of these 17 DEGs revealed notable interrelations among most of these genes (**Figure 4B**). These 17 genes were submitted to the STRING database and analyzed using Cytoscape 3.8.0 software for constructing a protein-protein interaction (PPI) network, which consisted of 17 nodes and 20 edges (**Figure 4C**).

### 3.4 Development and validation of a prognostic model for endometrial cancer based on diabetes-related genes

**Figure 5:**
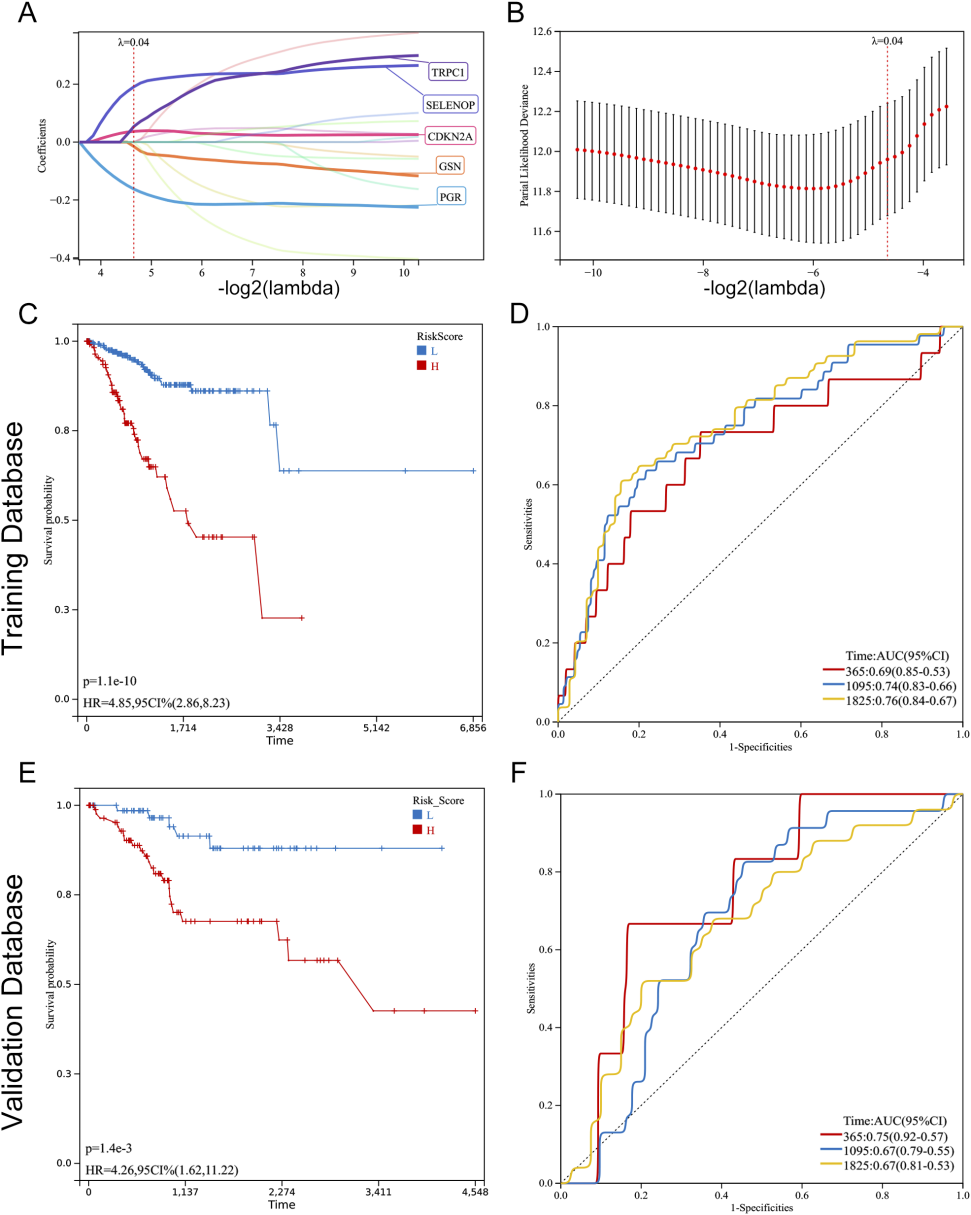
Construction and validation of a prognostic model based on diabetes-related genes in UCEC. **(A, B)** Lasso Cox regression analysis of 17 prognosis-related genes. **(A)** Coefficient path plot showing variable shrinkage with increasing lambda (optimal lambda = 0.0398). **(B)** Partial likelihood deviance plot identifying the optimal lambda (vertical line). Five genes (TRPC1, SELENOP, CDKN2A, GSN, PGR) were selected for the final model. **(C, E)** Kaplan-Meier survival curves comparing overall survival (OS) between high-risk and low-risk groups in the training **(C)** and validation **(E)** cohorts. Patients were stratified by the optimal risk score cut-off (0.1897). Significant OS differences were confirmed by log-rank test (training: P < 0.001; validation: P = 0.001). **(D, F)** Time-dependent ROC curves evaluating the model’s predictive accuracy for 1-, 3-, and 5-year OS in the training **(D)** and validation **(F)** cohorts. AUC values: training (1-year: 0.69, 3-year: 0.74, 5-year: 0.76; all P < 0.001); validation (1-year: 0.75, 3-year: 0.67, 5-year: 0.67; all P < 0.01).

In this investigation, Lasso regression analysis was utilized to ascertain five genes associated with prognosis: TRPC1, SELENOP, CDKN2A, GSN, and PGR. Among these, CDKN2A, SELENOP, and TRPC1 were identified as indicators of poor prognosis, while GSN and PGR were linked to favorable outcomes (**Figures 5A, B**). Based on the prognostic model, the optimal cut-off value was determined for the risk assessment. In the training cohort, the high-risk classification included 119 individuals, while the low-risk classification encompassed 258 individuals. In the validation cohort, the high-risk group consisted of 92 individuals, and the low-risk group included 71 individuals. Following this classification, we conducted a comparative analysis of the ROC curves and the survival outcomes of patients diagnosed with UCEC across both cohorts. The Kaplan-Meier survival analysis indicated that individuals within the low-risk category exhibited significantly superior overall survival (OS) compared to their high-risk counterparts, a trend that was evident in both the training cohort (P < 0.001) and the validation cohort (P < 0.01). This finding suggests that the risk score serves as a reliable prognostic indicator for EC patients (**Figures 5C, E**). Furthermore, the ROC curve analysis evaluated the predictive capacity of the risk model in both datasets, affirming the robustness and accuracy of the prognostic model predicated on diabetes-associated genes (**Figures 5D, F**).

### 3.5 Association of diabetes-related genes with risk stratification and survival outcomes in EC patients

**Figure 6:**
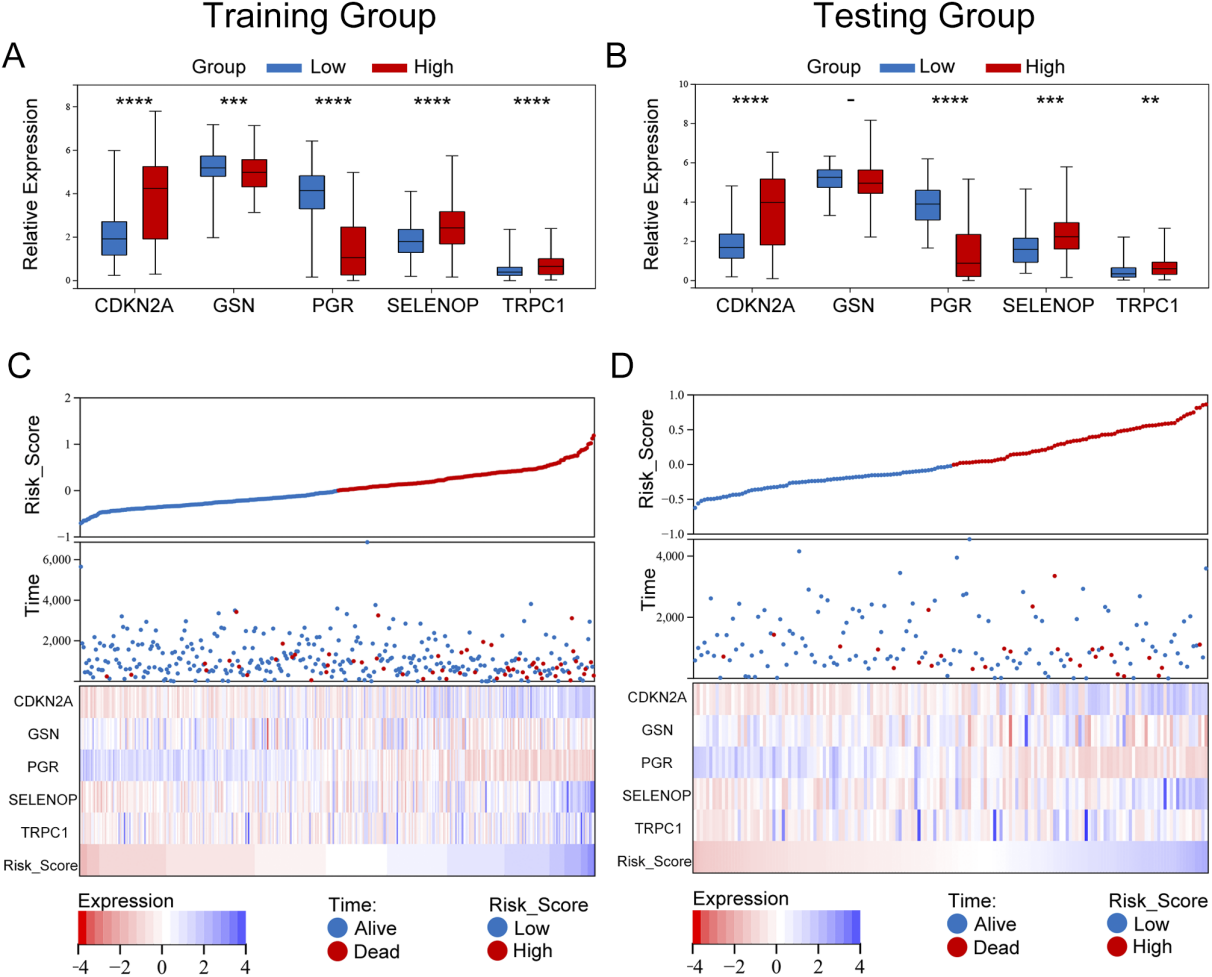
Expression differences and risk stratification of model genes in UCEC patients. **(A, B)** Box plots comparing the expression of five prognostic genes (TRPC1, SELENOP, CDKN2A, GSN, PGR) between high-risk and low-risk groups in the training **(A)** and validation **(B)** cohorts. Significance was determined by the Wilcoxon rank-sum test (****P < 0.0001, ***P < 0.001, **P < 0.01). Notably, GSN and PGR were significantly upregulated in the low-risk group, while CDKN2A, SELENOP, and TRPC1 were elevated in the high-risk group. **(C, D)** Integrated visualization of risk scores, survival outcomes, and gene expression heatmaps for the training **(C)** and validation **(D)** cohorts. Patients were stratified by the optimal risk score cut-off (0.1897), with high-risk patients (red) showing higher mortality rates. The heatmap confirmed consistent expression patterns across cohorts, with high-risk patients exhibiting elevated CDKN2A/SELENOP/TRPC1 and low-risk patients showing dominant GSN/PGR expression.

In our model, we identified five differentially expressed diabetes-related genes, with GSN and PGR exhibiting heightened expression levels in the low-risk group, whereas CDKN2A, SELENOP, and TRPC1 demonstrated increased expression in the high-risk group. The findings from both the training and validation cohorts were largely consistent (**Figures 6A, B**). We undertook a comparative analysis of the risk score distributions, survival statuses, and heatmaps between the training and validation sets for EC patients (**Figures 6C, D**), which revealed no notable differences between the two cohorts. The risk curves and scatter plots for both sets indicated a positive correlation between mortality and risk scores. The heatmaps for the three classifications confirmed the elevated expression of GSN and PGR in the low-risk group, while demonstrating increased levels of CDKN2A, SELENOP, and TRPC1 in the high-risk group.

### 3.6 Validation of key gene protein expression in endometrial tissue using the HPA database

**Figure 7:**
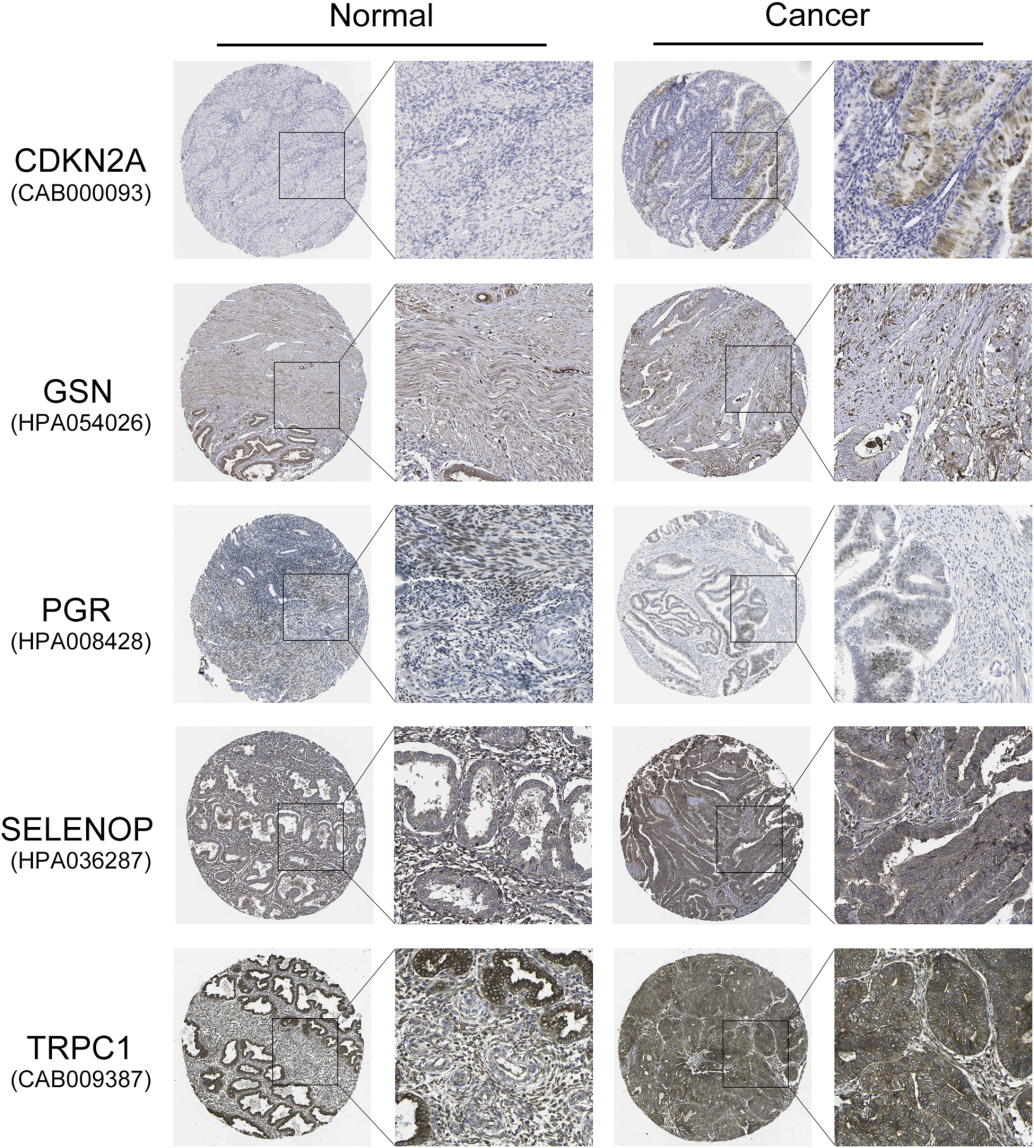
Validation of protein expression levels of five key diabetes-related genes in EC and normal endometrial tissues using the HPA database. Representative IHC staining images show the protein expression of *CDKN2A*, *GSN*, *PGR*, *SELENOP*, and *TRPC1* in normal endometrial tissues and endometrial carcinoma tissues. *CDKN2A*, *SELENOP*, and *TRPC1* exhibit higher protein expression in EC tissues compared to normal tissues, whereas *GSN* and *PGR* show lower expression in EC tissues.

To further validate the protein expression levels of the five key diabetes-related genes (CDKN2A, GSN, PGR, SELENOP, and TRPC1) identified in our prognostic model, we utilized IHC staining data from the HPA database. As shown in **Figure 7**, IHC images revealed that CDKN2A, SELENOP, and TRPC1 were expressed at higher levels in endometrial carcinoma tissues compared to normal endometrial tissues, consistent with their mRNA upregulation in high-risk patients. Conversely, GSN and PGR exhibited lower protein expression in tumor tissues relative to normal tissues, aligning with their favorable prognostic roles and reduced expression in the high-risk group. These findings not only corroborate our transcriptomic analysis but also reinforce the potential biological significance of these genes in endometrial cancer progression.

### 3.7 Experimental validation of key gene expression by quantitative PCR

**Figure 8:**
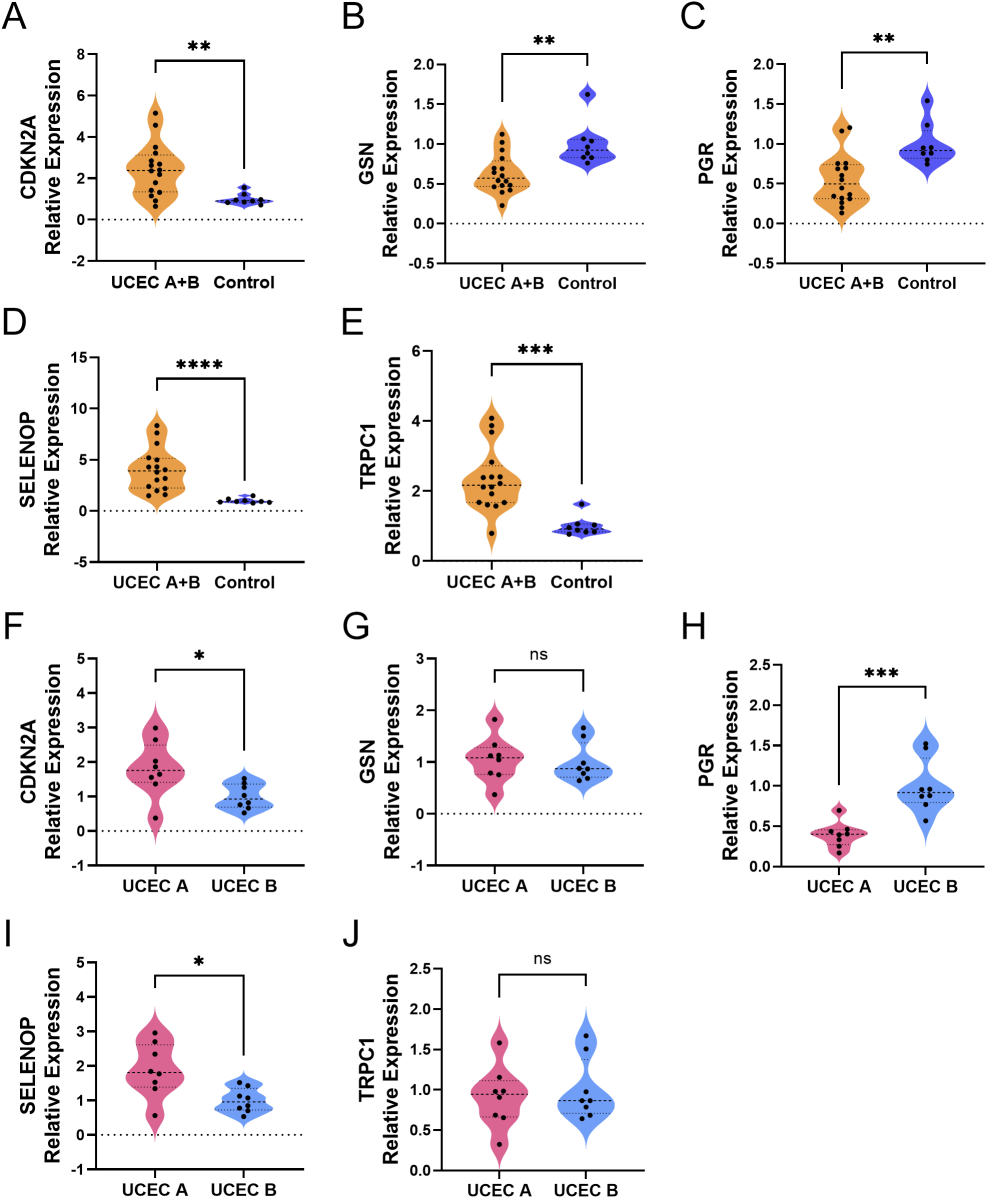
Differential expression of diabetes-related genes in UCEC tissues. **(A–E)** Comparison of gene expression between the combined UCEC group (UCEC A + UCEC B, n=16) and the Control group (normal endometrium, n=8): CDKN2A **(A)**, GSN **(B)**, PGR **(C)**, SELENOP **(D)**, TRPC1 **(E)**. **(F–J)**. Direct comparison of gene expression between UCEC patients with diabetes (UCEC A, n=8) and those without diabetes (UCEC B, n=8): CDKN2A **(F)**, GSN **(G)**, PGR **(H)**, SELENOP **(I)**, TRPC1 **(J)**. Statistical significance: ns > 0.05; *P < 0.05; **P < 0.01; ***P < 0.001; ****P < 0.0001.

To molecularly validate the expression patterns of the five key genes (TRPC1, SELENOP, CDKN2A, GSN, and PGR) identified from our prognostic model, we performed qRT-PCR on clinical tissue samples. The mRNA expression levels of TRPC1, SELENOP, and CDKN2A were significantly upregulated, while GSN and PGR were significantly downregulated in UCEC tissues compared to normal controls (p < 0.05) (**Figures 8A–E**). Furthermore, stratification of UCEC patients by diabetic status revealed that the dysregulation of SELENOP, CDKN2A, and PGR was specifically exacerbated by diabetes. The expression levels of SELENOP and CDKN2A were significantly higher, while PGR was significantly lower in diabetic UCEC patients than in non-diabetic patients (p < 0.05). In contrast, TRPC1 and GSN expression showed no significant difference between these two subgroups (**Figures 8F–J**). These results not only confirm the protein-level findings from the HPA database but also establish SELENOP, CDKN2A, and PGR as key genes whose expression is specifically modulated by the diabetic milieu.

### 3.8 The personalized prognostic prediction model showed robust predictive accuracy

**Figure 9:**
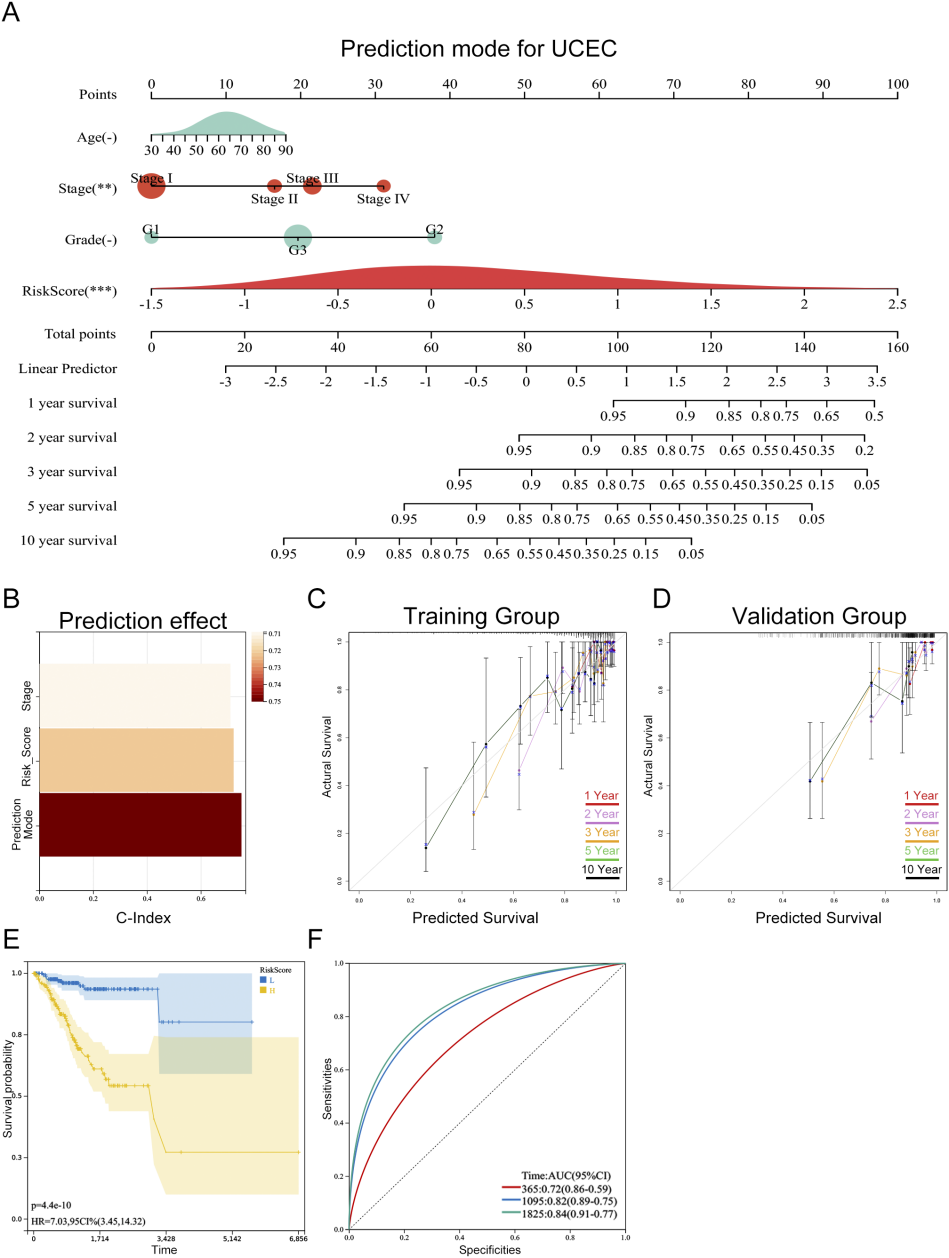
Personalized prognostic model for progression-free survival (PFS) in UCEC patients. **(A)** Nomogram predicting 1-, 2-, 3-, 5-, and 10-year PFS probabilities based on risk score and clinical parameters. The asterisks (-, **, ***) next to each variable denote its statistical significance in the multivariate Cox model (-P > 0.05, **P < 0.01, ***P < 0.001). **(B)** Model performance assessed by C-index (0.781, 95% CI: 0.72–0.84, P = 1.1e-19), indicating excellent discriminative ability. **(C, D)** Calibration curves for training **(C)** and validation **(D)** cohorts, showing alignment between predicted and observed PFS (Hosmer-Lemeshow test P > 0.05, no significant deviation). **(E)** Kaplan-Meier survival curves stratified by nomogram-predicted risk groups, with significant PFS difference between high-risk and low-risk patients (log-rank test P < 0.001). **(F)** Time-dependent ROC curves for 1-, 3-, and 5-year PFS prediction, with AUC values of 0.72, 0.82, and 0.84, respectively (all P < 0.001).

To enhance the clinical applicability of our prognostic prediction model, we developed a personalized prediction framework that integrates key variables such as patient age, histological stage, grade, and risk score. As shown in **Figure 9A**, this personalized model effectively estimates survival probabilities at 1, 2, 3, 5, and 10 years for patients with endometrial cancer. The nomogram model boasts a C-index of 0.781 (95% CI: 0.72-0.84), with a p-value of 1.1e-19, reflecting its robust predictive performance (**Figure 9B**). Calibration curves derived from both training and validation datasets exhibit a strong alignment between the nomogram’s predictions and actual outcomes, highlighting its exceptional predictive accuracy (**Figures 9C,D**). By stratifying the TCGA-UCEC dataset into high-risk and low-risk groups based on the median risk score from the nomogram, Kaplan-Meier survival analysis revealed a significantly more favorable prognosis for the low-risk group compared to the high-risk group (**Figure 9E**). Furthermore, ROC curve analysis for the high-risk and low-risk groups demonstrated area under the curve (AUC) values of 0.72, 0.82, and 0.84 for 1-year, 3-year, and 5-year survival, respectively, underscoring the model’s discriminative ability (**Figure 9F**).

### 3.9 Biological processes and pathways enriched for the hub genes

**Figure 10:**
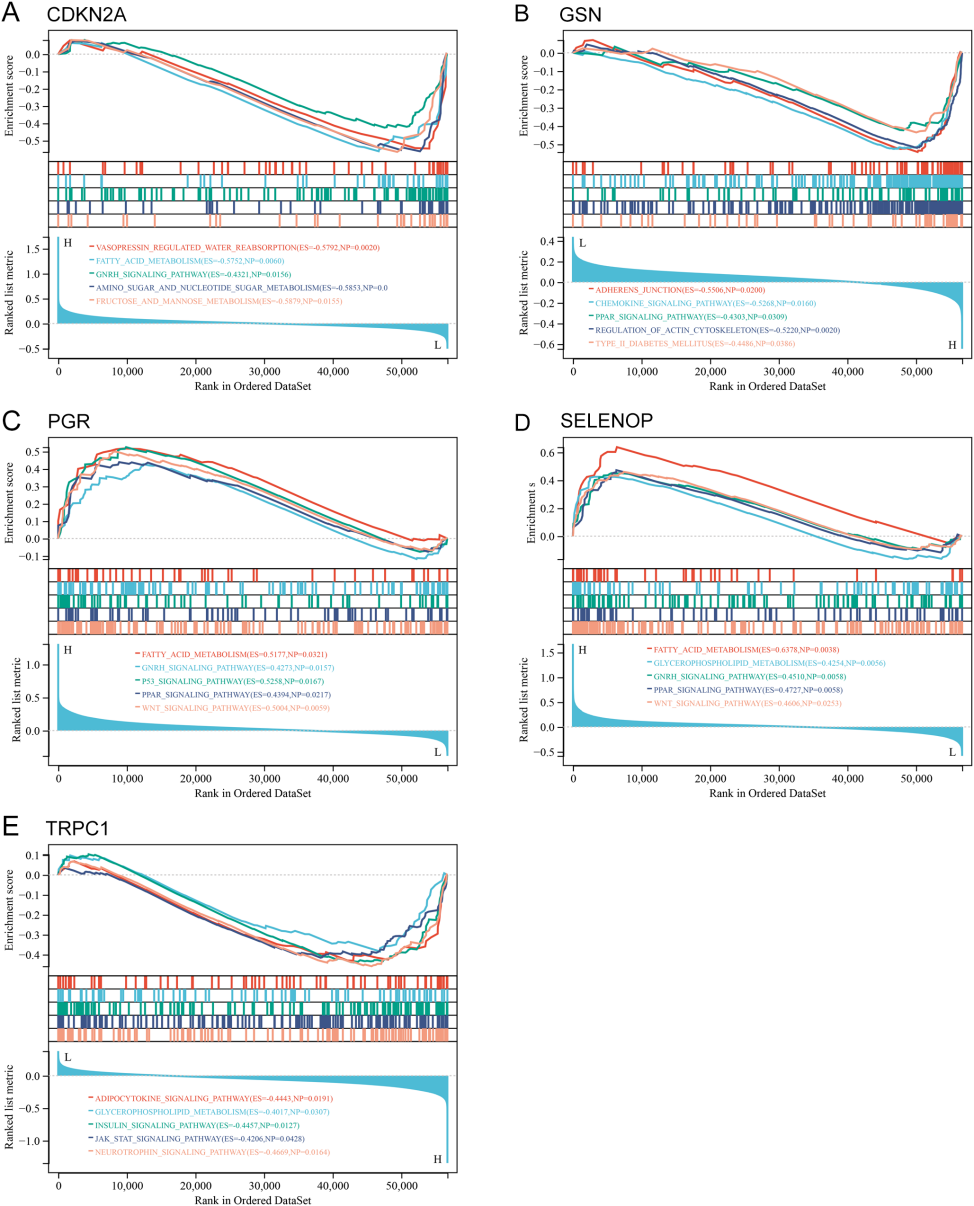
GSEA of hub genes in UCEC. **(A-E)** GSEA plots showing enriched pathways for each hub gene: **(A)** CDKN2A, **(B)** GSN, **(C)** PGR, **(D)** SELENOP, **(E)** TRPC1. P value of < 0.05 and FDR of < 0.25.

To investigate the potential biological roles of these genes, we performed GSEA. The results revealed significant enrichment in pathways concerning glucose and lipid metabolism (PPAR_SIGNALING_PATHWAY, FATTY_ACID_METABOLISM, INSULIN_SIGNALING_PATHWAY), cellular membrane structure (GLYCEROPHOSPHOLIPID_METABOLISM), embryonic development and tissue homeostasis (WNT_SIGNALING_PATHWAY), regulation of sex hormones (GNRH_SIGNALING_PATHWAY), and inflammatory responses (ADIPOCYTOKINE_SIGNALING_PATHWAY) (**Figure 10**; **Supplementary Table S5**).

### 3.10 Analysis of risk score correlation with tumor microenvironment, immunity, and somatic mutations

**Figure 11:**
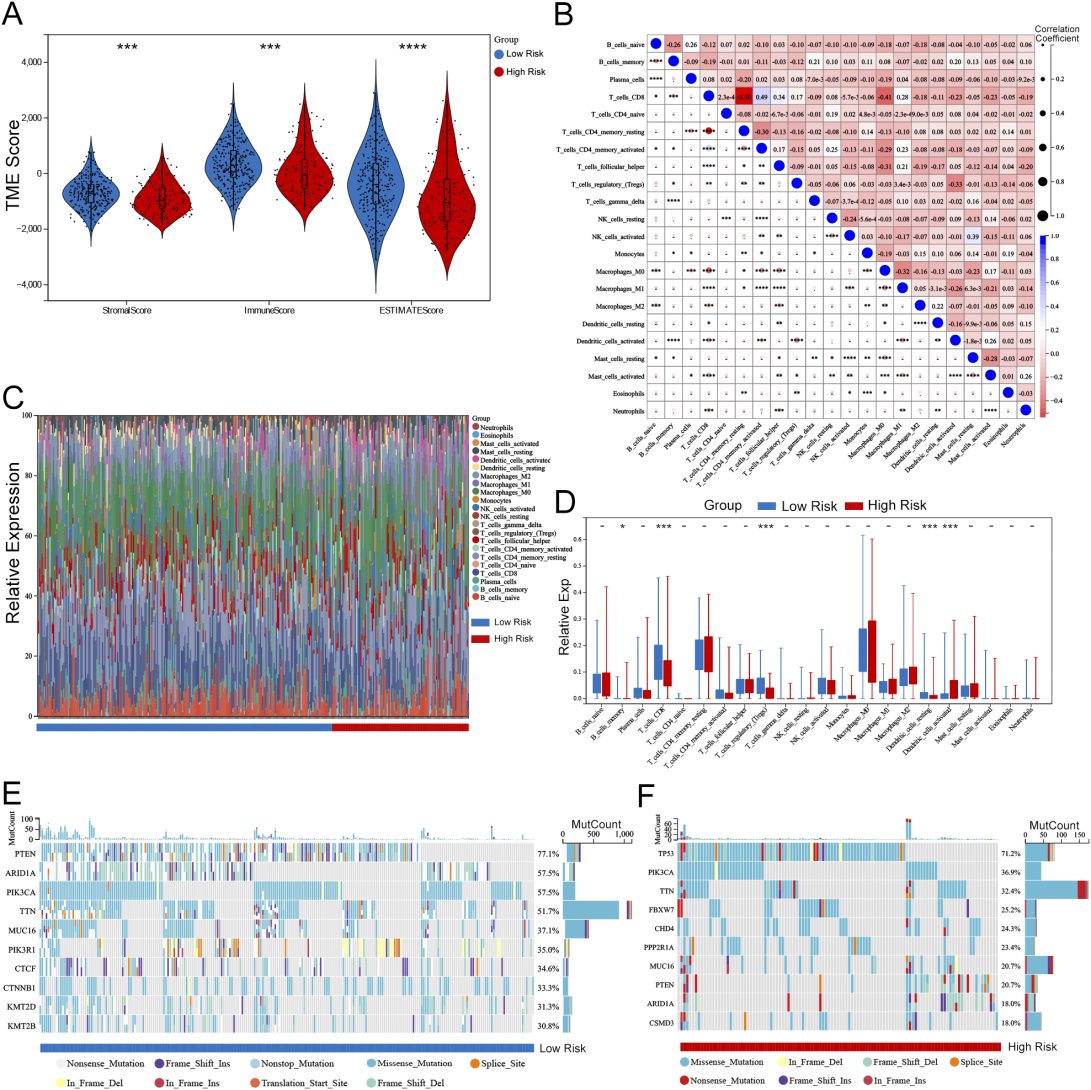
Association between risk score and TME in UCEC. **(A)** Violin plots comparing StromalScore, ImmuneScore, and ESTIMATEScore between high-risk and low-risk groups (Wilcoxon test, P < 0.001). **(B)** Correlation matrix of 22 immune cell populations (Pearson correlation, significant correlations marked with *P* < 0.05). **(C)** Stacked bar plots showing the distribution of immune cell types in high-risk vs. low-risk groups (e.g., CD8 T cells enriched in low-risk group, *P* = 0.002). **(D)** Box plots highlighting differential expression of key immune cells (e.g., M1 macrophages: *P* < 0.01; Tregs: *P* < 0.05). **(E, F)** Waterfall plots of somatic mutations in low-risk **(E)** and high-risk **(F)** groups. TP53 mutation frequency was higher in high-risk group (71.2% vs. 32.5%, Fisher’s exact test P < 0.001). Statistical significance: -P > 0.05, *P < 0.05, **P < 0.01, ***P < 0.001, ****P < 0.0001.

The assessment of the TME underscores its pivotal role in either immune suppression or activation, which is crucial for cancer prevention and therapeutic strategies. The pathway enrichment analysis indicated a relationship with immunity between the two risk groups. Considering the influence of gene transcripts (GTs) in the TME, we applied an estimation algorithm to derive scores for individual samples within the high-risk and low-risk groups. Our results demonstrated that the ImmuneScore, ESTIMATEScore, and StromalScore were all significantly elevated in the low-risk group in comparison to the high-risk group (**Figure 11A**). The TME, comprising immune cells, extracellular matrix, inflammatory cytokines, and diverse growth factors, plays a substantial role in determining clinical treatment responses and diagnostic outcomes. The CIBERSORT algorithm was utilized to estimate the proportions of 22 immune cell types in UCEC samples from 258 individuals in the low-risk group and 119 in the high-risk group, as depicted in histograms (**Figure 11C**). The results of the correlation analysis for these immune cell types are outlined (**Figure 11B**), and a comparative examination of immune cell expression profiles across the low-risk and high-risk groups is presented through box plots (**Figure 11D**). Additionally, we conducted an analysis of somatic mutations in both risk groups, revealing the top ten genes with the highest mutation frequencies, illustrated through waterfall plots (**Figures 11E, F**). Notably, the gene exhibiting the highest mutation frequency within the high-risk group was TP53, which accounted for 71.2% of the total mutations.

## 4 Discussion

UCEC (19, 20) is a prevalent gynecological cancer that significantly challenges women’s health worldwide. The incidence of UCEC is increasing. It is closely linked to metabolic disorders like diabetes (5, 6). Diabetes may worsen cancer development through various biological pathways and negatively impact patient prognosis (21–23).Consequently, researching the interaction between diabetes-related factors and the molecular mechanisms of UCEC has become a key area of focus. A deeper understanding in this field is expected to drive the development of more effective therapeutic strategies and improve prognostic models for patients.

Utilizing data from TCGA, this study identified 186 DM-DEGs in UCEC within the context of diabetes. Previous research has indicated that metabolic dysregulation in diabetes is associated with altered gene expression in cancers, including UCEC (24, 25). Functional enrichment analysis revealed that DM-DEGs play a pivotal role in cellular stress response and proliferation regulation, processes that are crucial for tumor initiation and progression. In particular, DM-DEGs were enriched in key metabolic pathways such as the PPAR signaling pathway, which is vital for glucose and lipid metabolism, and its dysregulation may promote tumorigenesis (26, 27). The PI3K-Akt signaling pathway and central carbon metabolism pathways in cancer were also significantly enriched, indicating that metabolic dysregulation plays a key role in the pathogenesis of UCEC under diabetic conditions (28, 29). Moreover, alterations in the insulin signaling pathway, a hallmark of type 2 diabetes, were associated with the aggressive behavior of UCEC, suggesting that therapeutic interventions targeting this pathway may be particularly important for diabetic patients. Additionally, the study found that the abnormal activation of the Wnt signaling pathway, which is related to cancer progression (30, 31), also operates in diabetes-associated UCEC. These findings underscore the pivotal role of molecular crosstalk in the diabetes-UCEC link, pinpointing targets for innovative therapeutic development to enhance patient outcomes. The study elucidates their complex interplay and introduces a prognostic model incorporating clinical variables, offering a novel approach to personalized care.

We identified five key genes—*CDKN2A*, *SELENOP*, *GSN*, *PGR*, and *TRPC1*—as significant biomarkers in the prognostic model for patient stratification. Notably, the expression patterns of these genes in high-risk patients revealed distinct pathogenic insights. *CDKN2A*, a classic tumor suppressor gene that regulates the cell cycle by inhibiting cyclin-dependent kinases (32, 33), was surprisingly upregulated in our high-risk UCEC group. This finding aligns with the model wherein its overexpression signifies cellular senescence or a compensatory response to oncogenic stress, a mechanism potentially exacerbated by metabolic dysfunction. *SELENOP* (34), a selenium transport protein critical for antioxidant defense, was also upregulated in high-risk patients. Elevated *SELENOP* levels, linked to both cancer progression and diabetes, may promote tumor growth by modulating inflammation and oxidative stress. *TRPC1*, a calcium channel associated with cell proliferation (35), was upregulated in the high-risk group, suggesting its role in enhancing tumor invasiveness and therapeutic resistance.

To further validate the protein expression levels of the identified key genes in endometrial tissue, we utilized IHC staining data from the HPA database. The HPA database provided IHC images of endometrial cancer and normal endometrial tissues, allowing us to confirm the differential expression of *CDKN2A*, *SELENOP*, *GSN*, *PGR*, and *TRPC1* at the protein level. This validation step is crucial as it bridges the gap between transcriptomic data and protein expression, reinforcing the biological significance of our findings. Critically, IHC staining confirmed strong accumulation of *CDKN2A*/p16 protein in tumor tissues, which aligns with its mRNA upregulation in high-risk patients. While *CDKN2A* is a tumor suppressor, its robust protein expression is a recognized biomarker of oncogene-induced cellular senescence or a dysfunctional cell cycle checkpoint in many cancers, a state that could be exacerbated by the diabetic metabolic environment. Similarly, *SELENOP* was overexpressed in tumor tissues, where it may promote tumor growth through increased inflammation and oxidative stress. In contrast, the reduced expression of *GSN* and *PGR* in tumor tissues supports their role as favorable prognostic markers, potentially through impaired cell adhesion and hormone response, respectively. Lastly, *TRPC1* was upregulated in endometrial carcinoma, potentially enhancing tumor cell proliferation and invasive capabilities through its regulation of calcium signaling and crosstalk with hormone pathways. These protein-level observations significantly bolster the clinical relevance of our prognostic model.

To experimentally validate these findings, we further investigated the expression patterns of these five key genes in clinical tissue samples. Our analysis revealed a distinct stratification: while *TRPC1*, *SELENOP*, *CDKN2A*, *GSN*, and *PGR* all showed significant dysregulation in UCEC tissues compared to normal endometrium, only *SELENOP*, *CDKN2A*, and *PGR* exhibited diabetes-specific modifications in UCEC patients. Specifically, we observed significant further suppression of *PGR* and enhanced upregulation of *SELENOP* and *CDKN2A* in diabetic UCEC tissues. These results strongly suggest that the diabetic milieu, particularly hyperinsulinemia and associated oxidative stress, actively remodels the molecular landscape of UCEC by specifically enhancing progesterone receptor loss while amplifying stress-response and senescence pathways. In contrast, expression levels of *TRPC1* and *GSN* remained comparable between diabetic and non-diabetic UCEC subgroups, indicating their involvement in general carcinogenesis rather than diabetes-specific pathways.

We validated the predictive performance of the risk score model using ROC analysis and Kaplan-Meier survival curves. Significant differences in OS were observed between high-risk and low-risk groups in both the training and validation cohorts, with p-values of 1.1 × 10^-10 and 0.001, respectively. This underscores the model’s effectiveness in stratifying patient risk. This is consistent with previous studies that established the prognostic value of gene expression profiles in various cancers, including endometrial cancer (36, 37). The AUC values for 1-year, 3-year, and 5-year survival outcomes were 0.72, 0.82, and 0.84, respectively, further confirming the accuracy of the model. Additionally, the calibration curve showed a high degree of concordance between predicted and observed outcomes, which reinforces the reliability of our nomogram. In summary, our study not only identified key biomarkers associated with diabetes and UCEC but also established a validated prognostic model that aids in clinical decision-making. Integrating diabetes-related DEGs into the prognostic framework offers a new perspective on the interplay between metabolic disorders and cancer prognosis, a field warranting further exploration in future research.

This study revealed significant differences in immune cell infiltration between high-risk and low-risk UCEC patients, as evidenced by the CIBERSORT algorithm and metrics like ImmuneScore, ESTIMATEScore, and StromalScore. These findings underscore the pivotal role of the TME in shaping immune responses and influencing prognosis (38). The observed discrepancies in immune scores between high-risk and low-risk groups not only delineate tumor biological characteristics but also likely influence immune responses. This aligns with existing research demonstrating how the TME affects tumor behavior and patient survival outcomes, highlighting the importance of robust immune responses in tumor suppression and enhanced prognosis (39). The relationship between immune scoring and risk stratification further emphasizes the vital role of immune regulation in cancer progression and treatment response (40, 41). Notably, the differential expression of CD8 T cells, Tregs, and dendritic cells suggests new avenues for developing immunotherapeutic strategies aimed at bolstering anti-tumor immunity and improving clinical outcomes for UCEC patients. For instance, enhancing CD8 T cell infiltration and activity while modulating Treg function could potentially tip the balance toward more effective tumor immune surveillance and destruction.

Moreover, our findings align with previous research indicating that metabolic dysfunction-related genes can accelerate EC progression not only by directly affecting tumor cells but also by modulating the tumor immune microenvironment (42). The TME, including infiltrating immune cells, plays a crucial role in shaping tumor development, therapeutic resistance, and clinical outcomes. Our observation that patients in the low-risk group exhibited higher TME scores and more diverse immune cell infiltration further supports the notion that a more active immune response is associated with a better prognosis. The enrichment of immune-related pathways in the low-risk group, as indicated by our pathway enrichment analysis, further strengthens this association.

Importantly, the observed differences in immune infiltration between high-risk and low-risk UCEC patients, as reflected by the immune scores, may also shed light on the interplay between diabetes and cancer, as chronic inflammation and immune dysregulation are common features of both conditions. In diabetes, immune cells can contribute to a pro-tumor microenvironment by promoting metabolic dysregulation, angiogenesis, and fibrosis. For instance, immune cells in diabetes can foster a pro-tumor microenvironment by augmenting the production of inflammatory cytokines, which can fuel tumor growth and angiogenesis, and modify immune checkpoint expression, thereby suppressing anti-tumor immunity. This suggests that the immune alterations observed in UCEC may be further exacerbated in the context of comorbid diabetes, highlighting the need to consider diabetes status when interpreting immune scores and developing therapeutic strategies.

These findings suggest that immune scores could be used to stratify UCEC patients and identify those who may benefit from specific immunotherapies, particularly those with comorbid diabetes. Specifically, exploring the mechanisms by which immune cells interact with metabolic pathways in the context of UCEC could unveil novel targets for therapeutic intervention, potentially leading to improved outcomes for patients with this aggressive form of cancer. In conclusion, while our study primarily focuses on the impact of diabetes-associated genes on UCEC, the immune score provides valuable insights into the tumor’s immune landscape, which may have broader implications for understanding the interplay between diabetes, inflammation, and cancer. Future research should further investigate the complex relationship between diabetes, immune infiltration, and cancer progression to develop more effective therapeutic strategies for patients with both conditions.

This study has several limitations that should be acknowledged. First, the analysis primarily relies on publicly available datasets such as TCGA, which predominantly represents the American population. This introduces potential biases related to sample collection, processing, and annotation, and limits the generalizability of the findings to other geographical regions or populations with distinct clinical characteristics. Although we employed stratified random sampling and 10-fold cross-validation to construct and validate the prognostic model, these approaches may not fully eliminate the potential heterogeneity inherent in the dataset. Therefore, future research should focus on incorporating data from diverse ethnicities, regions, and clinical backgrounds—particularly including patients with confirmed diabetes status—to enhance the model’s robustness and broader applicability.

Second, the absence of an independent external validation cohort weakens the robustness of the diabetes-related gene expression model. While internal validation methods were utilized, external validation remains essential for confirming the model’s predictive accuracy across different populations. Moreover, the study was based solely on gene expression data, without functional validation of the identified genes or their roles in diabetes-associated tumorigenesis.

Third, due to the lack of detailed clinical information regarding diabetes status in the TCGA cohort, we were unable to directly investigate the causal links between diabetes itself, diabetes-related genes, and cancer outcomes. This limitation is particularly evident in two aspects: 1) Mutation analysis: Although we stratified somatic mutations by risk score and observed significantly higher mutation frequencies of genes such as TP53 in the high-risk group, we did not explore whether these mutation differences are directly related to diabetes status. The absence of diabetes annotations prevented us from assessing causal relationships between mutational profiles and diabetes, rendering current conclusions indirect and speculative. Future studies should integrate clinical cohorts with well-documented diabetes information, combining genomic and metabolic data to explore the potential roles of diabetes-related mutations in endometrial cancer pathogenesis. 2) Biological mechanism inference: While enrichment analysis revealed that diabetes-associated differentially expressed genes (DM-DEGs) are significantly involved in biological processes such as glucose and lipid metabolism and insulin signaling pathways, the lack of definitive diabetes status data prevents direct validation of whether these gene expression changes are driven by diabetes or linked to its pathophysiological mechanisms. Thus, our conclusions should be considered preliminary and exploratory. Future prospective cohort studies incorporating clinical samples and molecular subtyping from diabetic patients are needed to clarify the true roles of DM-DEGs within the diabetes–endometrial cancer axis.

Finally, the cross-sectional nature of the data limits the ability to infer causal relationships between diabetes-related genes and endometrial cancer outcomes. Further longitudinal studies are necessary to elucidate these associations. Additionally, larger sample sizes through multi-center collaborations, particularly including patient data stratified by diabetes status, will be crucial to validate the generalizability and clinical utility of the model.

## 5 Conclusion

This study identified and characterized DM-DEGs in UCEC using TCGA data. Through LASSO-COX regression analysis, we developed a robust prognostic model based on five key DM-DEGs—*TRPC1*, *SELENOP*, *CDKN2A*, *GSN*, and *PGR*—that effectively stratifies patients into high- and low-risk groups. Our analysis of the tumor microenvironment and immune infiltration revealed potential for personalized treatments, underscoring the importance of metabolic disorders in cancer progression. Although the study is limited by the lack of diabetes status annotations in the TCGA cohort, which restricts the ability to directly establish causal links between diabetes-related mutations and cancer outcomes, it still provides novel insights into the complex interplay between diabetes and endometrial cancer. The DM-DEGs and associated pathways identified in this study—such as the PPAR and insulin signaling pathways—not only enhance our understanding of how diabetes influences UCEC pathogenesis but also lay a solid foundation for developing individualized therapeutic strategies for patients with both diabetes and endometrial cancer.

In summary, despite some conclusions being preliminary due to the indirect nature of current data, this work offers important evidence supporting the role of diabetes-related genes in endometrial cancer and sets the stage for future mechanistic and translational studies. We anticipate that subsequent research will build upon these findings to further validate their clinical applicability and broaden their implications.

## Data Availability

Publicly available datasets were analyzed in this study. The datasets of uterine corpus endometrial carcinoma (UCEC) analyzed in this study were sourced from the Cancer Genome Atlas (TCGA), accessible at https://cancergenome.nih.gov/.
